# Comparison of three instruments (compass, digital caliper and 3d software) for dental linear measurements on plaster and digitalized models of infants and children: an agreement and reproducibility study

**DOI:** 10.1590/2177-6709.30.6.e2525164.oar

**Published:** 2026-02-09

**Authors:** Patricia NADELMAN, Eduardo Otero Amaral VARGAS, Camila Silva de AMORIM, Amanda Cunha Regal de CASTRO, Matheus Melo PITHON, Lucianne Cople MAIA

**Affiliations:** 1 Federal University of Rio de Janeiro, Department of Pediatric Dentistry and Orthodontics, School of Dentistry (Rio de Janeiro/RJ, Brazil).

**Keywords:** Dental model, Dental impression technique, Digital technologies, Deciduous teeth, Modelos dentários, Técnica de moldagem dentária, Tecnologias digitais, Dentes decíduos

## Abstract

**Objective:**

The aim of the study was to evaluate the reproducibility and agreement of dental linear measurements made directly on plaster models with a compass, and a digital caliper, compared to digital measurements made through software on the same plaster models after digitalization.

**Methods:**

40 plaster models from 20 infants and children aged between 1 and 5 years old, of both sex, with complete or incomplete primary dentition, with or without premature loss of primary anterior teeth were selected. The models were scanned using the Optical 3D scanner. Two calibrated operators performed dental linear measurements with a compass and a digital caliper directly on the plaster models and with the Autodesk Meshmixer software on the digital models. Six measurements were evaluated: missing tooth space (if any), arch perimeter, arch length, arch width, intercanine length and intercanine width. Statistical analysis was performed through the Jamovi program. Inter-rater reproducibility was calculated using the intraclass correlation coefficient (ICC) and agreement between instruments was analyzed using the Bland-Altman test.

**Results:**

Inter-rater ICC showed excellent consistency, ranging from 0.93 to 1.00. Agreement between instruments was good, with the mean difference ranging from 0.034 mm (limits: -1.077 to 1.145 mm) to -1.002 mm (limits: -0.831 to 0.185 mm).

**Conclusion:**

Dental linear measurements performed on digital models obtained from scanned plaster models demonstrated excellent reproducibility and good agreement compared to those made with a compass and a digital caliper. All measurement instruments can be reliably used in dental clinical practice.

## RESUMO

**Objetivo:**

Este estudo teve como objetivo avaliar a reprodutibilidade e a concordância de medições dentárias lineares realizadas diretamente em modelos de gesso, utilizando um compasso e um paquímetro digital, em comparação com as medições digitais obtidas por meio de *software* nos mesmos modelos após digitalização.

**Métodos:**

Foram selecionados 40 modelos de gesso de 20 lactentes e crianças, com idades entre 1 e 5 anos, de ambos os sexos, com dentição decídua completa ou incompleta, com ou sem perda precoce de dentes decíduos anteriores. Os modelos foram escaneados utilizando um scanner óptico 3D. Dois operadores treinados e calibrados realizaram as medições dentárias lineares com compasso e paquímetro digital diretamente nos modelos de gesso, e com o *software* Autodesk Meshmixer (versão 3.5.474) nos modelos digitalizados. Foram avaliadas seis medidas: espaço decorrente da ausência dentária (quando presente), perímetro do arco, comprimento do arco, largura do arco, comprimento intercaninos e largura intercaninos. Os dados foram tabulados e a análise estatística foi realizada com o programa Jamovi (versão 2.2). A reprodutibilidade interexaminadores foi calculada por meio do coeficiente de correlação intraclasse (ICC), e a concordância entre os instrumentos foi analisada pelo teste de Bland-Altman.

**Resultados:**

O ICC interexaminadores demonstrou consistência excelente, variando de 0,93 a 1,00. A concordância entre os instrumentos foi considerada boa, com a média das diferenças variando de 0,034 mm (limites: -1,077 a 1,145 mm) a -1,002 mm (limites: -2,632 a 0,627 mm).

**Conclusão:**

As medições dentárias lineares realizadas em modelos digitais obtidos a partir da digitalização de modelos de gesso apresentaram excelente reprodutibilidade e boa concordância, em comparação com aquelas realizadas com compasso e paquímetro digital. Todos os instrumentos de medição podem ser utilizados com segurança na prática clínica odontológica.

## INTRODUCTION

The management of developing dentition and occlusion in Pediatric Dentistry is crucial to enable the proper growth and development of child’s oral cavity, impacting speech, eating, and the correct alignment of permanent teeth.^1^ Primary teeth act as natural space maintainers of permanent teeth, guiding them into their correct positions during eruption.^1^ If a primary tooth is lost prematurely, adjacent teeth can shift, causing the permanent tooth to erupt in the wrong place.^1^

Considering the importance of primary dentition, periodical dental occlusion examination is essential to verify possible abnormalities in tooth development and growth pattern, either for individuals with premature loss of primary teeth or for those without losses.^1^ Complementary exams may be necessary to complete the diagnosis verified during the clinical exam and among them, the analysis of study plaster models may be highlighted.^2^

Acquisition of plaster models from dental arches impression has been considered the gold standard and the most used technique of dental arch reproduction.^3^ It captures morphological conditions of patients’ occlusion in a specific time; complementing the diagnoses and establishing treatment plan; and also enabling an analysis of clinical cases evolution.^4^ Besides that, the possibility of digitalizing plaster models, or even directly scanning dental arches, has become a common resource in dental clinic routine.^5^

Regardless of the type of study model - plaster or digital -, dimensional changes in arches are evaluated through dental linear measurements related to dental arches development.^6^ Traditionally, these measurements were performed using a compass or a brass wire. Later, digital caliper was introduced and currently computer software and digital models are the latest tools used.^7^

In order to assist pediatric dentists in deciding the best measurement instrument for dental clinic, a comparison between all of them, specifically for dental linear measurements in primary arches, becomes necessary. Therefore, the aim of the present study was to evaluate reproducibility and agreement of dental linear measurements performed through software compared to compass with pointer on both sides and digital caliper on digital and plaster models of the same pediatric patients with or without premature loss of primary anterior teeth. The null hypothesis was that there was no differences between dental linear measurements obtained directly on plaster models with compass, and digital caliper, compared to digital measurements made through software on the same plaster models after digitalization. 

## METHODS

### STUDY DESIGN

The present study is part of a prospective cohort study. Research Ethics Committee approved the study protocol (approval number: 02502818.7.0000.5257, report number: 5.621.927) and an Informed Consent Form was provided to all of tutors allowing participation of infants and children in the study.

This is a study in which primary dentition arches of infants and children were measured to assess reproducibility and agreement of dental linear measurements made directly on plaster models with a compass, and a digital caliper, compared to digital measurements made through software on the same plaster models after digitalization in 3D optical scanner.

### PARTICIPANTS

Eligible participants who sought care at the Department of Pediatric Dentistry and Orthodontics of School of Dentistry from Universidade Federal do Rio de Janeiro composed this convenience sample. Recruitment period took place between April 2019 and March 2020.

Sample consisted of infants and children with primary dentition (complete or incomplete), from one to five years old, including both sex, that had premature lost or extraction of primary incisor(s) or canine(s) due to trauma and those without teeth losses. 

Exclusion criteria were: patients with special needs; premature loss or extraction of posterior teeth; teeth with cavitated caries lesions and/or restoration; non-nutritive habits; patients with orthodontic or orthopedic appliances; not meeting any of the inclusion criteria; or when the guardian refused to participate of the study. 

### TEST METHODS

Initially, infants and children’s upper and lower arches impressions were taken with alginate Orthoprint Tipo I (Zhermack, Badia Polesine, Italy) in standard perforated plastic (Maquira, Maringá, Paraná, Brazil and Morelli, Sorocaba, São Paulo, Brazil) or aluminum trays (Tecnodent, Indaiatuba, São Paulo, Brazil). Study models were created with special orthodontic plaster (Asfer Indústria Química Ltda, São Caetano do Sul, São Paulo, Brazil) in proportion recommended by the manufacturer, prepared and cut according to proposals from the literature.^8^

The plaster models were blindly and individually scanned using the optical 3D scanner (Open Technologies, Rezzato, Lombardy, Italy). Models scanning sequence consisted of scanning the upper model, then the lower model and, lastly, the occluded models in order to obtain inter-arches relation, as well as sagittal, vertical and cross-section adjustment of intercuspation.

Six dental linear measurements concerning dental arch development were considered:^9^ (1) missing tooth space (if any), (2) arch perimeter, (3) arch width, (4) arch length, (5) intercanine width and (6) intercanine length (Table 1). 

**Table 1:** Dental linear measurements used to evaluate occlusion development.

Variable	Definition	Illustration
(1) Missing tooth space	Determined by the distance between the interproximal surfaces of the teeth adjacent to the missing tooth.	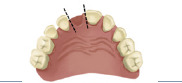
(2) Arch perimeter	Measurement of the arch from the distal midpoint of the primary second molar (or the last tooth present in the arch) from one side to the other side.	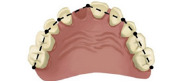
(3) Arch width	Distance between central fossae of occlusal surface of primary second molars or primary first molars, if those were not present.	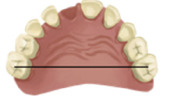
(4) Arch length	Perpendicular distance from contact point of central incisors to the line between the central fossae of the occlusal surfaces of primary second molars or primary first molars, if those were not present.	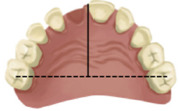
(5) Intercanine width	Distance between the cusp tips of primary canine.	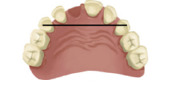
(6) Intercanine length	Perpendicular distance from contact point of central incisors to the line passing through the cusp tips of primary canines.	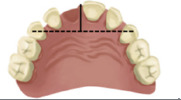

**Figure 1:**
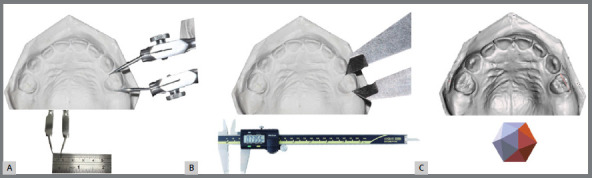
A) Compass measurements. B) Digital caliper measurements. C) Digital measurements.

Two trained and calibrated operators (P.N. and E.O.A.V., average ICC 0.97 and 0.99, respectively) performed the dental linear measurements, directly and digitally, in a blinded and independent way. Direct measurements were performed with one compass (Ice, São Paulo, Brazil) (Fig. 1A) and one digital caliper (Absolute Digimatic Caliper, Mitutoyo, Kawasaki, Japan) (Fig. 1B). Measurements from the compass were confirmed with a ruler (Acrimet®, São Bernardo do Campo, São Paulo, Brazil). Digital measurements were made using the Autodesk Meshmixer software (version 3.5.474, California, United States) (Fig. 1C). 

Samples were not measured with both instruments sequentially to avoid that one-instrument measurements influenced the others. All data were tabulated in individual tables for each operator.

## STATISTICAL ANALYSIS

Statistical analysis was performed using the Jamovi program, version 2.2 (Sydney, Australia). Intraclass correlation coefficient (ICC) was applied to calculate intra- and inter-rater reproducibility in each model type. Bland-Altman test was used to assess agreement of measurements’ comparison in plaster models and digital models between the two examiners. Mean difference between the instruments and the limits of agreement were used to explain data comparisons. A posteriori power analysis was performed in G*Power software,^10^ based on the mean and standard deviation of the differences between the digital caliper and software “tooth space loss” measurements, indicated an effect size of 0.68, and achieved power of 0.80.

## RESULTS

### PARTICIPANTS

Forty plaster models including maxillary and mandibular casts from 20 patients, aged between 1 year and 5 years old, of both sex (12 girls and 8 boys), were measured. Among these 20 patients, 11 showed premature loss of primary anterior teeth and 9 had no premature losses.

### TEST RESULTS

The mean intra-rater ICC values ​​were 0.97 for operator 1 and 0.99 for operator 2. These high ICC values suggest that the rater’s measurements are reliable and consistent over time.

Reproducibility among the operators revealed an excellent inter-rater ICC either for plaster models measurements with compass and digital caliper or for digital models measurements with software showing agreement values ranging from 0.93 to 1.00 (Table 2). The lowest agreement value was from the intercanine length of lower models measured with digital caliper. The highest agreement value resulted from digital measurements of intercanine width of upper models made with software.

**Table 2:** ICC reproducibility showing agreement values in dental linear measurements using compass, digital caliper and software instruments.

Variables	Dental arch	Compass		Digital caliper		Software	
		Agreement	Consistency	Agreement	Consistency	Agreement	Consistency
Tooth loss space	Upper	0.999	0.999	0.999	0.999	0.999	0.999
Arch perimeter	Upper	0.994	0.996	0.997	0.996	0.991	0.994
Arch width	Upper	0.987	0.992	0.993	0.988	0.996	0.996
Arch length	Upper	0.991	0.993	0.995	0.991	0.957	0.960
Intercanine width	Upper	0.999	0.999	0.999	0.999	1.000	1.000
Intercanine length	Upper	0.992	0.984	0.990	0.993	0.993	0.994
Tooth loss space*	Lower	-	-	-	-	-	-
Arch perimeter	Lower	0.998	0.998	0.998	0.999	0.998	0.998
Arch Width	Lower	0.988	0.986	0.986	0.990	0.972	0.972
Arch Length	Lower	0.994	0.989	0.989	0.993	0.984	0.983
Intercanine width	Lower	0.998	0.996	0.997	0.999	0.999	0.999
Intercanine length	Lower	0.963	0.928	0.933	0.961	0.930	0.943

* There is no value because there was no tooth loss in the lower arch.

**Figure 2:**
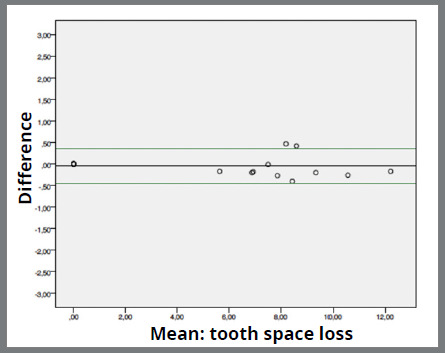
Bland-Altman plot showing the difference between digital caliper and software of “tooth space loss” measurements.

**Figure 3:**
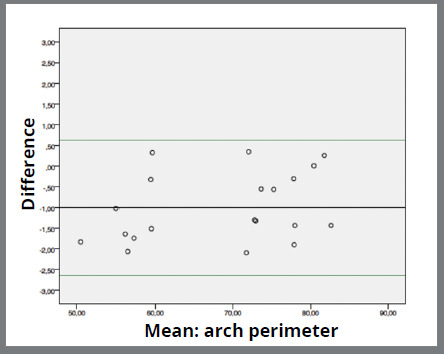
Bland-Altman plot showing the difference between digital caliper and software of “arch perimeter” measurements.

**Figure 4:**
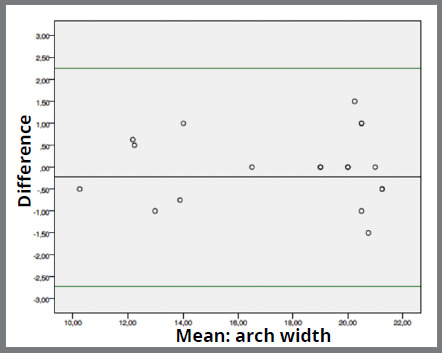
Bland-Altman plot showing the difference between compass and software of “arch width” measurements.

**Figure 5:**
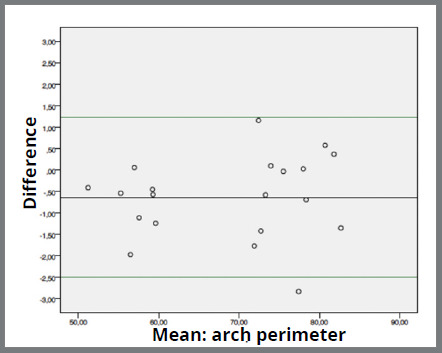
Bland-Altman plot showing the difference between compass and software of “arch perimeter” measurements.

Agreement between the instruments was good. Mean difference between digital caliper and software range from -0.048mm (0.207; 0.464) (Fig. 2) in “tooth loss space” measurement to -1.002mm (0.831; 0.185) (Fig. 3) in “arch perimeter” measurement. Mean difference between compass and software ranged from -0.231mm (1.269; 0.283) (Fig. 4) in “arch width” measurement to -0.633 (0.952; 0.213) (Fig. 5) in “arch perimeter” measurement (Table 3). 

**Table 3:** Bland-Altman values comparing the measurement instruments.

Dental linear measurement	Measurement instruments	Dental arch	n	Bias (mm)	Lower limit of agreement (mm)	Upper limit of agreement (mm)
Tooth loss space	Digital caliper x software	Upper	20	-0.048	-0.456	0.359
Arch perimeter	Digital caliper x software	Upper	20	-1.002	-2.632	0.627
Arch width	Digital caliper x software	Upper	20	0.258	-0.961	1.476
Arch length	Digital caliper x software	Upper	20	-0.199	-1.291	0.894
Intercanine width	Digital caliper x software	Upper	20	-0.109	-0.839	0.622
Intercanine length	Digital caliper x software	Upper	20	0.162	-1.101	1.424
Tooth loss space	Digital caliper x software	Lower	20	0.00	0.00	0.00
Arch perimeter	Digital caliper x software	Lower	20	-0.807	-2.246	0.631
Arch width	Digital caliper x software	Lower	20	0.287	-1.357	1.931
Arch length	Digital caliper x software	Lower	20	-0.185	-1.518	1.148
Intercanine width	Digital caliper x software	Lower	20	0.111	-0.701	0.922
Intercanine length	Digital caliper x software	Lower	20	0.065	-1.268	1.398
Tooth loss space	Compass x software	Upper	20	-0.163	-0.743	0.418
Arch perimeter	Compass x software	Upper	20	-0.633	-2.501	1.235
Arch width	Compass x software	Upper	20	0.034	-1.077	1.145
Arch length	Compass x software	Upper	20	-0.388	-1.593	0.818
Intercanine width	Compass x software	Upper	20	-0.060	-0.865	0.745
Intercanine length	Compass x software	Upper	20	0.044	-0.915	1.003
Tooth loss space	Compass x software	Lower	20	0.00	0.00	0.00
Arch perimeter	Compass x software	Lower	20	-0.228	-2.276	1.820
Arch width	Compass x software	Lower	20	0.034	-1.637	1.706
Arch length	Compass x software	Lower	20	-0.23	-2.72	2.26
Intercanine width	Compass x software	Lower	20	0.095	-0.608	0.799
Intercanine length	Compass x software	Lower	20	0.175	-0.844	1.194

## DISCUSSION

Management of the developing dentition and occlusion is an important component of pediatric dentistry routine.^11^ Guidance of the eruption and the development of primary dentition contribute for the early diagnosis of occlusion complications during childhood.^12^ It assists not only the prevention and treatment of malocclusions, but also support the normal growth of dental arches, jaws and face.^13^

During occlusion supervision, deviations in dental arches’ normal evolution can be noted such as premature loss of primary teeth.^14^ Sequels resulting from premature loss of primary anterior teeth may negatively affect eruption of permanent successor teeth;^15^ promote the establishment of malocclusion in permanent dentition^16^ and damage to primary arch integrity.^17^ As the consequences of premature loss of primary anterior teeth are controversial, poorly studied and evidence is outdated, it is important to monitor children’s occlusion development in order to evaluate the need to intervene.^18^

In this context, digital scanners and digital study models should be beneficial for pediatric dentists during the occlusion monitoring to avoid unnecessary treatments and costs.^13^ Considering technological progress, the introduction of digital models has become a reality in late 1990s.^5^ Since then, many studies have replaced dental plaster models by digital models to facilitate diagnosis and treatment plan.^19^ Following that, systematic reviews have shown that digital models are as reliable as plaster models, with high agreement and reproducibility.^3^ ^,^ ^7^ ^,^ ^20^

 Then, the purpose of the present study was to assess which measurement instrument would be the most advantageous for pediatric dentistry clinics. To achieve this, reproducibility and agreement of dental linear measurements were performed on digital models through software and plaster models both with compass and digital caliper. Agreement assesses how closely different instruments agree with each other in measuring the same object, while reproducibility is related to repetition. Thus, it is the degree to which measurements are repeated under the same conditions.^21^

Nevertheless, there is little scientific evidence published in the literature evaluating the application of digital models specifically for measurements in primary dentition.^13^ Such evidence would be important since the primary arch has different characteristics from the permanent arch, such as: number of teeth, arch dimensions, dental sizes, among others.^22^ Therefore, the present study aimed to assess reproducibility and agreement of dental linear measurements performed specifically on digital models of infants and pre-school children.

Every practitioner should assess and guide the occlusion during dental attendance toward optimal outcomes.^12^ It contributes not only for early diagnosis of occlusal changes, but also for treatment decisions regarding space management; use of space maintainers and other appliances; or even primary teeth extraction.^18^ Such management of developing occlusion can be done in infants and children through clinical examination and complementary exams, including study models analysis.^23^ To monitor the development of primary dental arches, the research included plaster models with primary dentition (complete or incomplete) from infants and children.

During the appropriate occlusion supervision, pediatric dentist may be faced with premature loss of primary anterior teeth, which also indicates the need for monitoring the emergence of dimensional arch changes because of this loss.^24^ Also, supervising children developing occlusion after premature loss of primary anterior teeth becomes essential since its sequels are still controversial, poorly studied and evidence is outdated.^25^ Besides the inclusion of models without any tooth/teeth loss, models of patients with premature loss of primary incisors and canines were also included for the dental linear measurements of the present study.

Dental linear measurement is derived when two reference points are connected to obtain dental sizes or distances in dental arches, for example.^12^ It is an important step in diagnosis procedure, treatment plan and definition of follow-up periods. Regarding linear measurements, the most used for dental arches evaluation are: tooth sizes or tooth loss space (if any), arch perimeter, arch width, arch length, intercanine width and intercanine length.^10^ ^,^ ^13^ These six dental linear measurements were used both for measurements in physical and digital study models.

Performing digital measurements through software has become a reality in dental offices^18^ since there are some disadvantages to the use of plaster models, such as: difficulties of storage, risk of fracture and physical damage in measuring procedure.^19^ Among advantages of using digital models, possibility of store and reuse created images in software and also share them with other clinicians or multidisciplinary departments can be highlighted.^20^

Regarding agreement, Bland-Altman test compared the three different measurement instruments - compass, digital caliper and software - to indicate if there was agreement between them. The result showed that agreement between instruments was good with mean difference ranging from 0.034mm (-1.077; 1.145) to -1.002mm (0.831; 0.185). The highest results show that mean difference between digital caliper and software range -1.002mm in arch perimeter. This fact can be understood by the method acquisition, since for the arch perimeter measurement, it is necessary to divide dental arch in six sectors, measuring them individually - molars, canine and incisors of the right hemi-arch, and molars, canine and incisors of the left hemi-arch - and then adding them up.

Reproducibility of quantitative measurements obtained by different operators was assessed through the inter-rater ICC test. The ICC is a parameter widely used in scientific research to measure correlation between evaluation samples between two or more evaluators when there is a quantitative variable. Koo and Li^26^ suggested that values less than 0.5 are poor, between 0.5 and 0.75 are moderate, between 0.75 and 0.9 are good, and greater than 0.9 are excellent. ICC results from the present study were considered excellent with values ranging from 0.93 to 1.00. It means that these instruments have the property of repeating similar results with different examiners.

Current findings corroborate results presented in most recent systematic reviews from scientific literature^3^ ^,^ ^7^ ^,^ ^20^ ^,^ ^27^ and, also provide additional information about the possibility of performing digital measurements made on plaster models after digitalization in pediatric dentistry clinic. But it is worth mentioning that the measurement instrument should be the one that best fits the reality of each dentist. Therefore, professionals need to evaluate advantages and disadvantages of each instrument in terms of value, acceptability by the patient, practicality, and most importantly, availability at the time of the appointment. 

## CONCLUSION

Based on the results obtained from this research, dental linear measurements performed in digital models obtained by scanning plaster model achieve excellent reproducibility and good agreement compared to compass and digital caliper. The present results allow the possibility of training dentists to perform dental linear measurements regardless of the instrument used. All measurement instruments should be safely used in dental clinical practice.

## References

[1] American Academy of Pediatric Dentistry (2016). Guideline on Management of the Developing Dentition and Occlusion in Pediatric Dentistry. Pediatr Dent.

[2] Ferreira JB, Christovam IO, Alencar DS, da Motta AFJ, Mattos CT, Cury-Saramago A (2017). Accuracy and reproducibility of dental measurements on tomographic digital models a systematic review and meta-analysis. Dentomaxillofac Radiol.

[3] Rossini G, Parrini S, Castroflorio T, Deregibus A, Debernardi CL (2016). Diagnostic accuracy and measurement sensitivity of digital models for orthodontic purposes A systematic review. Am J Orthod Dentofacial Orthop.

[4] Rischen RJ, Breuning KH, Bronkhorst EM, Kuijpers-Jagtman AM (2013). Records needed for orthodontic diagnosis and treatment planning a systematic review. PLoS One.

[5] Mok KH, Cooke MS (1998). Space analysis a comparison between sonic digitization (DigiGraph Workstation) and the digital caliper. Eur J Orthod.

[6] Moyers RE (1991). Ortodontia 4th ed. Rio de Janeiro: Guanabara Koogan;.

[7] Luu NS, Nikolcheva LG, Retrouvey JM, Flores-Mir C, El-Bialy T, Carey JP (2012). Linear measurements using virtual study models. Angle Orthod.

[8] Camargo ES, Mucha JN (1999). Moldagem e modelagem em Ortodontia. Rev Dental Press Ortod Ortop Facial.

[9] Padma Kumari B, Retnakumari N (2006). Loss of space and changes in the dental arch after premature loss of the lower primary molar a longitudinal study. J Indian Soc Pedod Prev Dent.

[10] Faul F, Erdfelder E, Lang AG, Buchner A (2007). G*Power 3 a flexible statistical power analysis program for the social, behavioral, and biomedical sciences. Behav Res Methods.

[11] Yousefi F, Shokri A, Zahedi F, Farhadian M (2021). Assessment of the accuracy of laser-scanned models and 3-dimensional rendered cone-beam computed tomographic images compared to digital caliper measurements on plaster casts. Imaging Sci Dent.

[12] McDonald RE, Avery DR, Dean JA (2011). Managing the developing occlusion In: McDonald and Avery's, ed. Dentistry for the child and adolescent. 9th ed. Maryland Heights, MO: Mosby/Elsevier;.

[13] Kaihara Y, Katayama A, Ono K, Kurose M, Toma K, Amano H (2014). Comparative analyses of paediatric dental measurements using plaster and three-dimensional digital models. Eur J Paediatr Dent.

[14] Nadelman P, Gárate KM, Oliveira A, Pithon MM, de Castro ACR, Maia LC (2021). Dental arch perimeter changes as a result from premature loss of primary anterior teeth due to trauma A case series in infant and pre-school children. Int J Paediatr Dent.

[15] Holan G, Ram D (1999). Sequelae and prognosis of intruded primary incisors A retrospective study. Pediatr Dent.

[16] Miyamoto W, Chung CS, Yee PK (1975). Effect of premature loss of deciduous canines and molars on malocclusion of the permanent dentition. J Dent Res.

[17] Clinch LM, Healy MJR (1959). A longitudinal study of the results of premature extraction of deciduous teeth between 3-4 and 13-14 years of age. J Contemp Dent Pract.

[18] Çayönü S, Demirel A, Sari S (2019). Should we use the digital models in pediatric dentistry. Cumhuriyet Dent J.

[19] Hajeer MY, Millett DT, Ayoub AF, Siebert JP (2004). Applications of 3D imaging in orthodontics Part II. J Orthod.

[20] Fleming PS, Marinho V, Johal A (2011). Orthodontic measurements on digital study models compared with plaster models a systematic review. Orthod Craniofac Res.

[21] Camardella LT, Souza JM, Villela BS, Villela OV (2014). Avaliação da acurácia e reprodutibilidade de modelos digitais por escaneamento do modelo de gesso. Ortodontia SPO.

[22] Quimby ML, Vig KW, Rashid RG, Firestone AR (2004). The accuracy and reliability of measurements made on computer-based digital models. Angle Orthod.

[23] Law CD (2013). Management of premature primary teeth loss in the child patient. J Calif Dent Assoc.

[24] Nadelman P, Bedran N, Magno MB, Masterson D, de Castro ACR, Maia LC (2020). Premature loss of primary anterior teeth and its consequences to primary dental arch and speech pattern A systematic review and meta-analysis. Int J Paediatr Dent.

[25] Nadelman P, Magno MB, Pithon MM, Castro ACR, Maia LC (2021). Does the premature loss of primary anterior teeth cause morphological, functional and psychosocial consequences. Braz Oral Res.

[26] Koo TK, Li MY (2016). A guideline of selecting and reporting intraclass correlation coefficients for reliability research. J Chiropr Med.

[27] Warnecki M, Nahajowski M, Papadopoulos MA, Kawala B, Lis J, Sarul M (2022). Assessment of the reliability of measurements taken on digital orthodontic models obtained from scans of plaster models in laboratory scanners A systematic review and meta-analysis. Eur J Orthod.

